# The Molecular Mechanisms of Anesthetic Action: Updates and Cutting Edge Developments from the Field of Molecular Modeling

**DOI:** 10.3390/ph3072178

**Published:** 2010-07-08

**Authors:** Edward J. Bertaccini

**Affiliations:** Department of Anesthesia, Stanford University School of Medicine, Co-Director of Operating Room and Intensive Care Services, Palo Alto VA Health Care System, 112A, PAVAHCS, 3801 Miranda Ave., Palo Alto, CA 94304, USA; E-Mail: edwardb@stanford.edu; Tel.: +1 (650) 493-5000; Fax: +1 (650) 852-3423

**Keywords:** anesthesia, molecular mechanism, molecular modeling, computational chemistry

## Abstract

For over 160 years, general anesthetics have been given for the relief of pain and suffering. While many theories of anesthetic action have been purported, it has become increasingly apparent that a significant molecular focus of anesthetic action lies within the family of ligand-gated ion channels (LGIC’s). These protein channels have a transmembrane region that is composed of a pentamer of four helix bundles, symmetrically arranged around a central pore for ion passage. While initial and some current models suggest a possible cavity for binding within this four helix bundle, newer calculations postulate that the actual cavity for anesthetic binding may exist *between* four helix bundles. In either scenario, these cavities have a transmembrane mode of access and may be partially bordered by lipid moieties. Their physicochemical nature is amphiphilic. Anesthetic binding may alter the overall motion of a ligand-gated ion channel by a “foot-in-door” motif, resulting in the higher likelihood of and greater time spent in a specific channel state. The overall gating motion of these channels is consistent with that shown in normal mode analyses carried out both *in vacuo* as well as in explicitly hydrated lipid bilayer models. Molecular docking and large scale molecular dynamics calculations may now begin to show a more exact mode by which anesthetic molecules actually localize themselves and bind to specific protein sites within LGIC’s, making the design of future improvements to anesthetic ligands a more realizable possibility.

## 1. Introduction: From Lipid to Protein Theories of Anesthetic Action

### 1.1. The Meyer-Overton and Lipid Theories of Anesthetic Action

For over 160 years, general anesthesia has been provided for the safety and comfort of a myriad of otherwise painful procedures, most notably surgery. Early investigations into the mechanisms of anesthetic action by Meyer and Overton [1] demonstrated a significant correlation of an anesthetic’s potency with its lipid solubility in olive oil. The inference from such studies was that anesthetics must act by altering the properties of a larger source of lipid in the body, the cell membrane. This became known as the Meyer-Overton Hypothesis. 

Since then, many studies have been performed to further characterize the more specific lipid environment in which anesthetics could act. These have included studies of anesthetic solubility in a variety of solvents [2,3]. Other studies have sought to elucidate the exact effect of an anesthetic on the characteristics of membrane bilayers. Some have shown significant effects of anesthetics on parameters such as membrane expansion [4], lipid order [5] and bilayer membrane lateral pressure profile [6]. While showing an anesthetic effect, the majority of these studies have, unfortunately, been carried out at anesthetic concentrations far beyond those that are clinically relevant. Furthermore, it is unclear the extent to which bilayer perturbations must occur in order to have a direct effect on ion channel conductance, the final common pathway of neuronal communications.

### 1.2. Exceptions to the Meyer-Overton Correlation

Until recently, the Meyer-Overton correlation of anesthetic potency with lipid solubility has remained a robust physical phenomenon. While the correlation is yet to be completely explained and accounted for, the original hypothesis stemming from that correlation now has some gross exceptions. Koblin and Eger [7,8] have demonstrated several classes of compounds that deviate from the Meyer-Overton rule. These include a multitude of polyhalogenated linear and cyclic hydrocarbons, whose lipid solubilities would predict reasonable anesthetic potency but whose actual anesthetic effect is slim to none. In particular, with regard to the minimum alveolar concentration (MAC) of anesthetic gas required to suppress motion to surgical incision in 50% of the patient population, these substances can be classified into those with partial anesthetic effect (the transitional compounds) and those with no effect at all (the nonimmobilizers). In fact, within the series of transitional anesthetic compounds, potency correlates with a compound’s hydrophilicity and not its lipophilicity. Other exceptions to this correlation include the differing potencies of various anesthetic stereoisomers despite similar lipophilic characteristics.

Since the advent of the anesthetic phenomenon, there have also been vast advances in the field of neurobiology. Many of the neuronal substrates responsible for the effects that anesthetics are meant to subdue have been worked out in great detail. It has become far more apparent that the mechanisms involved in the normal transduction of human neural activity lie in interneuronal synaptic communications. Furthermore, synaptic transmission has been painstakingly characterized as the summation of electrical activity from a wide array of membrane spanning ion channel proteins. However, to date there has been no direct proof that one can alter a lipid bilayer (such as by dissolving an anesthetic in it) in such a way as to have subtle but direct effects on membrane spanning proteins that are enough to grossly alter ion channel function and the subsequent behavior of a whole organism.

### 1.3. Direct Interactions of Anesthetics with Proteins

While hypotheses involving the direct effects of anesthetics on lipid bilayers indirectly affecting synaptic transmission have not been fully satisfactory, other investigations into the direct effect of anesthetics on proteins have proven more fruitful. Among the first to demonstrate the interaction of an anesthetic with a functional protein was the work of Franks and Leib [9,10]. They demonstrated the direct enzymatic suppression of firefly luciferase, the enzyme responsible for the light of the firefly, due to the formation of the anesthetic-protein complex. While luciferase may not have anything to do with the anesthetic effect, this was the first work showing an alteration in protein function as a result of its direct interaction with an anesthetic.

### 1.4. Altered Anesthetic Modulation of Ion Channel Electrophysiology via a Direct Effect

Many others have now gone on to show the electrophysiologic consequences of direct anesthetic binding to a variety of membrane-bound proteins actually associated with neuronal conductance. These include anesthetic effects on both voltage-gated (Na, K, Ca) and ligand-gated ion channels (glycine, GABA, AChR, serotonin, AMPA, kainate), as well as a variety of G-protein coupled receptors. In particular, the ligand-gated ion channels have been singled out because of their implication in the mediation of anesthetic-like states when interacting with other drugs (*i.e.*, the benzodiazepine and barbiturate effects on GABAR), as well as several key studies demonstrating how they are fundamentally affected by the presence of anesthetics. Mihic *et al*. [11] first demonstrated the role of specific amino acids necessary for the anesthetic effect on the human GlyRa1 in an oocyte preparation. They showed that mutation of serine 267 to a variety of other amino acids had significant bearing on the way in which anesthetics enhanced glycine-mediated chloride ion conductance. 

The question that originally arose from this work was whether the effect of their mutations involved the actual anesthetic binding site, or merely a related site that produced enough allosteric change to alter the anesthetic binding site. This issue was taken up by Mascia and colleagues [12] using a series of anesthetics derivatized with a methanethiosulfonate (MTS) moiety. The GlyRa1 could be mutated from serine to cysteine at the same 267 position noted above. This was shown to not significantly alter ion channel function. Then the mutated GlyRa1 was exposed to the anesthetic propanethiol, which produced a potentiating effect on ion conductance that was reversible upon washout. Next the mutated GlyRa1 was exposed to propanethiol in the presence of reducing agents that would catalyze the formation of a covalent disulfide bond between the cysteine at position 267 and the propanethiol. This produced a potentiating effect upon the ion channel that was of the same quality as that produced by the previous application of propanethiol alone, but it was irreversible even when all remaining reagent was removed from the system including the lipid bilayer. Both the same quality and magnitude of anesthetic effect was produced when the mutated system was exposed to propyl-MTS, the MTS derivative of the anesthetic propanethiol, forming the same disulfide bond more efficiently and without additional reducing agents. This demonstrated that, at least for the anesthetic propanethiol, it was both necessary and sufficient for the anesthetic to interact with the protein alone to produce its quantifiable effect. The mutated site was an actual anesthetic binding site and it was not necessary to implicate the lipid in the mechanism of action.

Finally Jurd and colleagues [13] have extended this result to demonstrate an effect on an entire organism. At the same homologous point in the mouse GABA beta 3 receptor, they produced a single point mutation that made mice completely insensitive to both propofol and etomidate, and partially insensitive to volatile anesthetics. While the result for the former two agents was quite dramatic, the effect of such a mutation on the modulatory capabilities of the volatile anesthetics was less well pronounced. In fact, the effect was only partial in nature at best, suggesting that the volatile anesthetics must have mechanisms that extend beyond a single ligand-gated ion channel effect, as noted above.

### 1.5. Studies of the Physicochemical Characteristics of an Anesthetic Binding Site

In order to better understand the possible physicochemical nature of the anesthetic binding site, many studies have been performed over the years that infer the possible characteristics of just such a site. Since the Meyer-Overton correlation still holds true for a great many anesthetic compounds, the anesthetic binding site must have some hydrophobic character. The exceptions to the Meyer-Overton rule do reveal, however, a dependence on hydrophilic characteristics within the series of transitional compounds [7]; therefore, there must be a way to introduce hydrophilicity into the anesthetic binding site. Johansson and colleagues [3] advanced solubility studies by demonstrating anesthetic potency correlations within a series of solvents that simulated various amino acid side chain environments. Their work pointed to a methionine-like solvent as one that best mimics an anesthetic binding site. It should be pointed out that methionine is intimately involved at the GABA beta 3 site mutated by Jurd *et al*. [13] Several others have implicated weak hydrogen bond types of interactions within any anesthetic binding site [14,15]. Trudell has studied the noble gases (*i.e.*, xenon) which have anesthetic effects and has demonstrated the correlation of anesthetic potency with the polarizability of the noble gases in a polar environment [16].

### 1.6. Reasons to Study Molecular Mechanisms of Anesthetic Action

Consequently, our group has sought to better elucidate a more exact mechanism of anesthetic action on the molecular and atomic scale. Such a detailed mechanistic understanding should have multiple benefits. First, paramount to any future anesthetic drug design will be the understanding of ligand-target site interactions at an atomic level. Second, a more thorough knowledge of the consciousness-altering effects of anesthetics may provide a greater insight into the actual neural, if not physicochemical, nature of consciousness itself. It is in this light that we have embarked on a two-pronged approach to studying the molecular mechanisms of anesthetic action. Our initial focus has been on the very detailed quantum mechanics analysis of anesthetic-protein interactions within several well characterized crystal structures of known atomic coordinates [17]. Secondly, because the proteins present in such currently available coordinate databases are simple globular structures not known to be involved in neural transmission, we have simultaneously used molecular graphics and modeling techniques to pursue our concurrent line of work: to build the first models of a putative anesthetic binding site within ligand-gated ion channels that are actually relevant to neuronal function [18].

## 2. Modeling A Putative Site of Anesthetic Action within Ligand-gated Ion Channels (LGICs): From Protein Schematic to Full-Scale Atomic Models

### 2.1. Physicochemical Basis of Anesthetic-Protein Interactions

Most anesthetics are of a variety of shapes and sizes but are not very large molecules. Therefore their overall binding motifs to proteins, in general, should be somewhat limited. In our initial studies, we set out to describe the general binding motifs of anesthetics to proteins in several well described anesthetic-protein complexes [17]. There have been many detailed studies of anesthetic-protein complexes that demonstrated direct binding of an anesthetic to a protein. They include bromoform-luciferase, halothane-albumin, halothane-cholesterol oxidase and dichloroethane-dehalogenase. More recently, bovine apoferritin has also been crystallized with halothane, isoflurane and a series of propofol-like derivatives [19,20]. Other less well defined crystallographic studies have included complexes of dichloroethane-insulin, [21] halothane-adenylate kinase, [22] and halothane complexed with rhodopsin [23]. Various synthetic four-helix bundles complexed with anesthetics [24] have also been studied by spectroscopic means. Additionally, several azi-alcohol derivatives have been used to characterize anesthetic binding sites within both protein-kinase C [25] and nAChR [26]. 

Based on our analysis of the aforementioned four high resolution crystal structures, [17] we have shown that the interaction of anesthetics with proteins involves a binding site characterized by several common hydrophobic and hydrophilic types of interactions. In fact, the binding pocket is amphiphilic in nature, involving both a variety of amino acid types as well as water. Such interactions involve weak hydrogen bonds, van der Waals contacts and other weak polar interactions that many previous studies have implicated as necessary for reversible anesthetic binding. In particular, such an amphiphilic binding site actually induces modest polarization of the anesthetic itself, thereby contributing what may be a significant part of the anesthetic binding energy. These general binding motifs, when applied to ion channel proteins that may be responsible for anesthetic effects, may help to explain the relative potencies of the transitional compounds, as well as those that obey the Meyer-Overton correlation.

Such an amphiphilic binding site is also consistent with the work of several other groups [27]. Recent analyses of several halogenated volatile anesthetics have described the necessary combination of both “bulk” properties and electrostatic interactions to adequately account for anesthetic binding and potency. In particular, Sewell and Sear [28,29] have used comparative molecular field analysis (CoMFA) to study the chemical characteristics of a large series of halogenated volatile anesthetics and have constructed an anesthetic pharmacophore that has both polar and nonpolar characteristics. This method is based solely on the analysis of ligands without reference to any specific protein binding site, yet reveals similar physicochemical characteristics for anesthetic binding. Also, Abraham *et al.* [15] have suggested that "general anesthetic target sites in animals must have, in addition to their overall hydrophobicity, a polar component which is a relatively poor hydrogen bond donor, but which can accept a hydrogen bond about as well as water." Additionally, Sandorfy [14] has concluded that weak H-bonds and van der Waals contacts are the prime mediators of reversible anesthetic-protein interactions. As can be seen in the next section, our most recent work on constructing molecular models of anesthetic binding sites in and about the transmembrane four-helix bundles of ligand-gated ion channels is highly consistent with this amphiphilic notion.

### 2.2 Defining the Structural Problem

**Figure 1:**
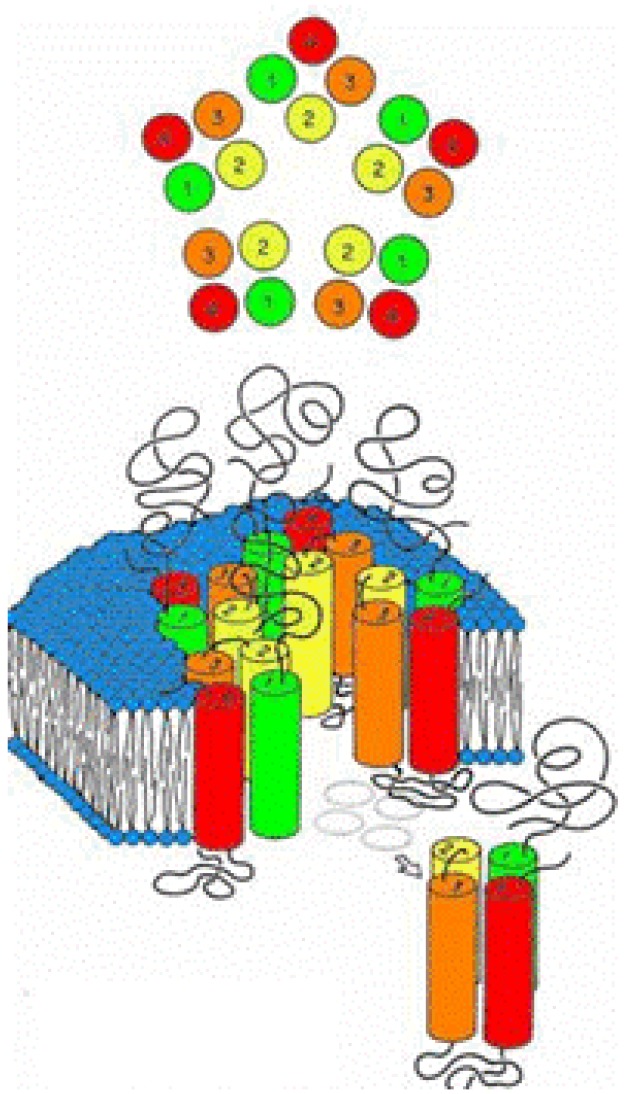
Original schematic structure of the family of ligand-gated ion channels, as revealed by low resolution cryoelectron micrographs showing the three-component of the protein, extracellular ligand binding domain (LBD), transmembrane domain TMD with cylindrical components number 1-4, and poorly defined intracellular domain.

The initial schematic structure of the family of ligand-gated ion channels, as revealed by low resolution cryoelectron micrographs, was that of a three-component protein (Figure 1) [30]. 

This was thought to be composed of a polar extracellular domain involved in native ligand binding (LBD), a hydrophobic transmembrane domain (TMD) where mutations alter anesthetic modulation, and a somewhat mysterious intracellular domain involved in various cytoplasmic interactions. An ion channel pore traverses this entire protein for the passage of ions specific to the channel type. Within this class of LGIC’s, there are both cationic and anionic conducting channels. To form these regions, the overall schematic called for the axially symmetric placement of five protein subunits into a pentameric arrangement around the central ion pore (Figure 1). Within each subunit, the transmembrane domains seemed to be composed of four individual “rod-like” regions. The questions at this point were many. What is the secondary structure of the transmembrane “rods”? How are these transmembrane domains within the individual subunits arranged? How are the individual subunits themselves arranged to form a transmembrane ion channel? Since mutations of amino acids within the transmembrane domains seem to alter anesthetic effects on these channels, where do the anesthetics actually bind?

For most proteins of interest, the typical sequence of study involves the purification of the protein material for crystallization and analysis by means of x-ray crystallography. This is especially important when one desires exact atomic coordinates for purposes of studying protein structure activity relationships and possible receptor-based drug design. However, the difficulty with the vast majority of membrane spanning proteins, especially the LGICs, lies in the destruction of relevant protein structure when removed from their native transmembrane environment.

This has prompted the use of computer-based theoretical techniques with the incorporation of a variety of experimental constraints, so as to build realistic, physically relevant models of the ion channel proteins within the family of LGICs. Specifically, with modern techniques of bioinformatics, computational chemistry, and molecular modeling, we have built models of the transmembrane subunit region within human glycine alpha one receptors (GlyRa1) and gamma amino butyric acid receptors (GABAR) that account for a large body of the physicochemical and experimental data that characterizes these proteins. [18,31,32,33,34,35,36,37,38] Most notably, these models contain cavities within the transmembrane region which demonstrate the proximity of amino acid residues known to be involved in the effects of anesthetics on these ion channels. These cavities form a plausible anesthetic binding site that is amphiphilic in physicochemical character and is large enough to contain an anesthetic [17,38].

### 2.3. Predicting Transmembrane Secondary Structure

Our initial work concerned the nature of the “rod-like” structures that composed the transmembrane tetrameric portion of the individual protein subunits [36]. To study this, the sequences of several human LGICs were obtained from the National Center for Biotechnology and Information. We utilized 10 state-of-the-art bioinformatics techniques to predict the transmembrane topology of this tetrameric region. The resulting sequences were aligned using the multiple sequence alignment method known as ClustalW [39,40]. The secondary structure predictions from each of the 10 topology prediction algorithms were superimposed onto the aligned amino acid sequences to analyze for consistency of prediction. Together, the consensus of these techniques predicted that the transmembrane subunits of the pentameric LGIC family were tetrameric bundles of alpha helices.

### 2.4. Building the Transmembrane Four-Helix Bundle and the Convergence of Residues Relevant to Anesthetic Binding

The next step was to use the principles of homology modeling in order to find a protein with a known three-dimensional structure and that looked in some way similar (either by sequence, protein fold or both) to the sequence and predicted fold of the LGICs [18,33,35]. Once such a protein of known structure was found, it could function as a template over which the sequence of the LGICs with unknown structure could be draped, with the subsequent transfer of three-dimensional coordinates to the new amino acid sequence. Therefore, the information regarding the alpha helical nature of the transmembrane rods was combined with the amino acid sequence information of various LGICs using the SeqFold algorithm [41] to search for modeling templates within a database of proteins with known three dimensional coordinates derived from the national Research Collaboratory for Structural Biology (RCSB, http://www.rcsb.org). 

**Figure 2:**
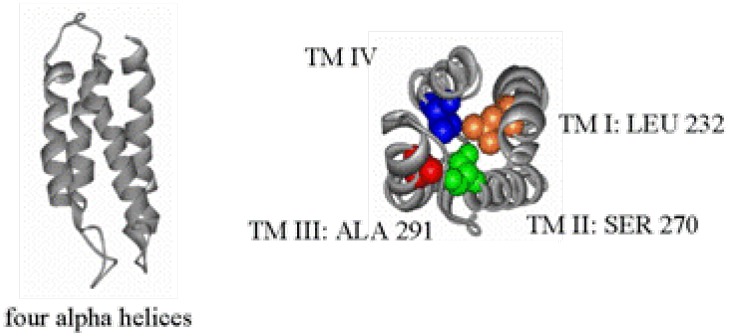
The Structure of the Four-helix Bundle Transmembrane Domain within the GABA alpha one receptor.

The SeqFold algorithm identified a relatively high-scoring modeling template in the coordinates of chain C of a bovine cytochrome oxidase (PDB ID 2OCC). This is a four-helix bundle of the up-down topology. The sequence of our LGIC was then aligned with this template using multiple scoring criteria. Manual refinement of the alignment closed sequence gaps to produce agreement with experimental labeling studies that had been carried out by many groups on the homologous receptors of the superfamily. Since the large intracellular cytoplasmic loop between TMD 3 and 4 had little resemblance to any template, the model was then edited so as to contain an abbreviated version of the loop composed of only nine native residues from the loop region itself along with a chain of five glycine residues substituted for the central remaining bulk of the loop. Structural assignment and refinement was achieved using the Modeler program [42]. The final structure demonstrated a cavity within the core of a four-helix bundle. Residues known to be involved in modulating anesthetic potency converge on and line this cavity. This would suggest that the binding sites for volatile anesthetics in the LGIC's are the cavities formed within the core of transmembrane four-helix bundles (Figure 2).

### 2.5 Building the Entire Transmembrane Domain

**Figure 3:**
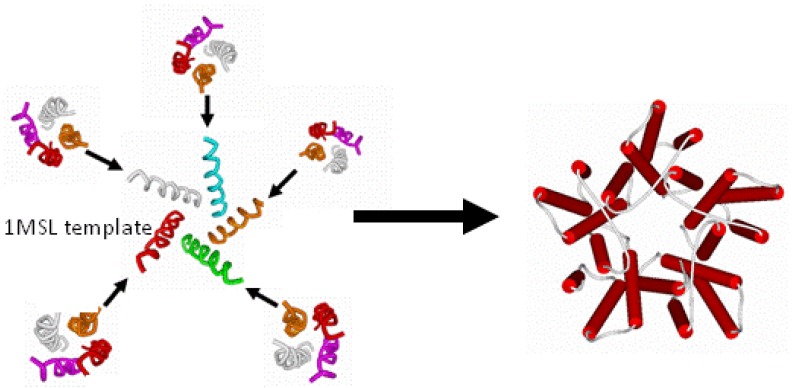
Merging the Subunits to Form the Entire Transmembrane Domain.

Once the four-helix bundle nature of the individual transmembrane domains had been identified, the subunit models had to be combined into their pentameric arrangement, so as to produce the entire transmembrane domain of the ion channel. For this purpose, a new template for a different form of alignment involving only the pore lining helices was found in the analogous bacterial mechanosensitive channel (PDB ID 1MSL). Only the channel-lining helices were used as the pentameric template around which to organize the channel-lining helices of our LGIC tetrameric subunit, so as to build a pentamer of tetramers for the entire TMD construct (Figure 3).

### 2.6. Building the Ligand-Binding Domain (LBD)

For the extracellular LBD, a homology model was built based upon the acetylcholine binding protein (AChBP, PDB ID 1I9B) of Sixma and coworkers [43]. Here, the sequence of a given LGIC was draped over the AChBP template with known 3D coordinates and energy optimized. In particular, the heteromeric GABAR is composed of two alpha subunits, two beta subunits and one gamma subunit. The determination of the unique clockwise orientation of these subunits around the central ion pore was made possible for the first time by the alignment of specific residues known to bind various ligands within the extracellular domain, most notably those residues involved in benzodiazepine binding. This clockwise orientation arranged the subunits in the order of gamma, alpha, beta, alpha, beta around the ion channel pore. 

### 2.7. Aligning and Merging the TMD to the LBD

**Figure 4:**
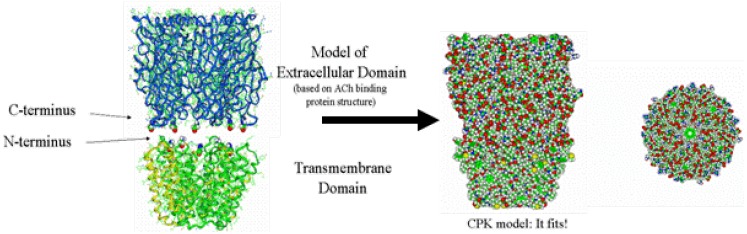
The Combination of the Extracellular Ligand-binding Domain with the Transmembrane Domain. Note the excellent tapered fit based on both outside protein perimeters and pore lining walls. Note also the contiguous ion channel pore traversing the entire protein complex.)

The LBD and TMD were then mated (Figure 4), according to distance restraints imposed by mutational data, with a surprisingly good fit, thereby producing the entire LGIC complex minus its intracellular cytoplasmic domain [37,38].

**Figure 5:**
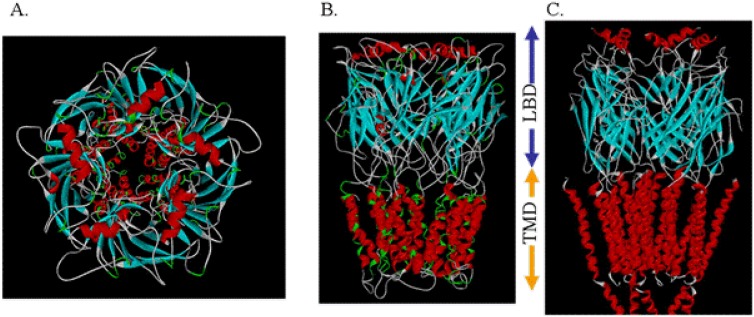
The Top View (A) and Side View (B) of one model of a ligand-gated ion channel. Note the similarity to the intermediate resolution cryoelectron micrograph of Unwin (C). LBD = ligand binding domain; TMD = transmembrane domain).

Using molecular mechanics forcefields, hydrogens were then added to the homology model structure and an automated conformation check performed to obtain a low energy state of the amino acid side-chains. After initially tethering the protein backbone, the entire complex underwent a series of restrained optimizations to eliminate any additional high energy conformations. In the end, final energy optimization was obtained without restraints and without gross distortion of tertiary structure. This produced a final construct that was reasonably similar to the intermediate resolution structure of the homologous nAChR derived from cryoelectron microscopy (Figure 5).

## 3. A Newer Alternative Model Based on Updated Prokaryotic Templates Changes the Putative Location of the Anesthetic Binding Site

**Figure 6:**
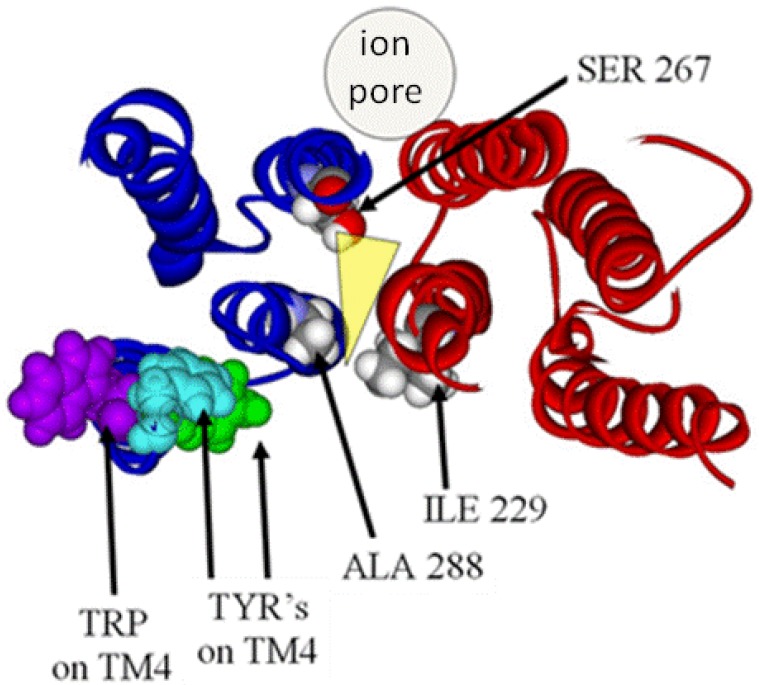
Magnified slab view of a GlyRa1 model in solid ribbon representation (blue and red for each subunit) showing residues involved in modulating anesthetic effect between two subunits. Note the location of the TM4 residues known to be relevant to anesthetic effect and their great distance from any putative intersubunit anesthetic binding site (transparent yellow wedge).

Recently, the X-ray structure of a pentameric ion channel from the prokaryotic *Erwinia chrysanthemi* (ELIC) has been crystallized at 3.3Å resolution [44,45,46]. The significant similarity of its amino acid sequence with the LGICs now allows for a reasonably robust homology model construct of the GlyRa1 in a way that does not require the formation of separate models with their subsequent merge into one final construct. Using these templates, newer models of a GlyRa1 were initially based on the prokaryotic pentameric ion channel in the closed state from ELIC [47]. This was followed by a GlyRa1 model based on the open state structures of a new homologous ion channel from the prokaryote *Gloebacter violaceus* (GLIC) in the open state [48]. These latter templates are relevant since anesthetics are thought to bind to and stabilize the open state of the GlyRa1. The 3D coordinates of two forms of GLIC (3EHZ and 3EAM) were obtained from the RCSB database. A BLAST sequence search was performed using the GLIC sequences. Among the best scored homologous human sequences were those of the GlyRa1. The sequence of the human GlyRa1 was obtained from the NCBI database. The template structures and the sequence of GlyRa1 were aligned with Discovery Studio 2.0.1 (Accelrys, San Diego, CA, USA) and the Modeler module was used for assignment of coordinates for aligned amino acids, the construction of possible loops, and the initial refinement of amino acid sidechains. The resulting BLAST derived scores suggested a close homology between the LGICs, GLIC and ELIC. Subsequent CLUSTALW alignment of the GLIC and GlyRa1 sequences demonstrated reasonable sequence similarity. The final model of the GlyRa1 from these templates is also a homomer with pentameric symmetry about a central ion pore and shows significant transmembrane alpha helical and extracellular beta sheet content, very similar to the models noted above. The model based on the ELIC template showed more of a closed channel state, while the model based on the GLIC templates showed a continuously open pore with a partial restriction within the transmembrane region. Three of the residues notable for modulating anesthetic action are on transmembrane segments 1-3 (TM1-3) (ILE229, SER 267, ALA 288). They now line the *inter*subunit interface (Figure 6), in marked contrast to the *intra*subunit binding site of our previous models (Figure 4). However, residues from TM4 that are known to modulate a variety of anesthetic effects on this or homologous LGICs cannot directly interface with any intersubunit anesthetic binding site (Figure 6). 

## 4. Results and Analysis of the LGIC Model: The Quandary of the Location of the Anesthetic Binding Site

Our current models of human GlyRa1 and GABARa1 homomers have demonstrated several key findings that are common amongst them. The extracellular LBD has a large component of beta sheet structure between the subunits of which may occur native ligand binding. This LBD mates extremely well with a model of the TMD built by completely independent means in one study. The transmembrane domain is composed of a pentamer of tetrameric alpha helical bundles, each associated with a putative anesthetic binding site. This site illustrates the convergence of residues known to be involved in anesthetic effects on these ion channels [11,34]. The size of the pore channel is in a range large enough to accommodate ion passage. The residues involved in the ion selectivity filter are located around the distal end of the TMD pore [49]. While our models were developed from completely theoretical means, the overall protein construct can be seen as extremely similar to the most recent model of the nicotinic acetycholine receptor (2BG9.) within torpedo californicus derived from the revised cryoelectron microscopy of Nigel Unwin [50] (Figure 1). Furthermore, both models can account for several of the residue positions known to be labeled by hydrophilic labels as well as those for similar experiments with hydrophobic labeling reagents [51,52,53] from studies involving the GlyRa1 and homologous positions within other LGIC’s. Both models can account for the results from cysteine modification studies showing irreversible anesthetic effects with methanethiosulfonate reagents introduced for TM2 and TM3 [12].

However, the model with the *intra*subunit binding site can readily account for the results from specific cysteine cross-linking studies between TM2 and 3 [54]. This model also gives a clear means by which TM4 mutations could directly impinge on an anesthetic binding site, as suggested by Jenkins [55,56] and Harris [57].

Alternatively, the model with the *inter*subunit anesthetic binding site also has some unique and valuable characteristics. There have been many others who have suggested the intersubunit location for a putative anesthetic binding site, but the vast majority of these were based upon the somewhat intermediate resolution structure of the nAChRa1 from cryoelectron microscopy [26,58,59,60,61,62]. Our most recent model involving an intersubunit anesthetic binding site is the result of what many would claim to be a more robust modeling method based on a more reliably refined template. Additionally, data from a different set of crosslinking studies involving cysteine mutations between TM1 and TM3 as well as TM2 and TM3 [60,61] are more reliably accounted for with this model and not by that containing the intrasubunit binding site. The difficulty with this model, however, lies in its inability to account for any direct effects of mutations on TM4 with regards to their alterations of ion channel sensitivities to anesthetics. Any model containing an intersubunit anesthetic binding site necessarily positions TM4 at a great distance from such a site. This is a quandary that will only be resolved by further mutational analyses and/or the final generation of actual LGIC crystallography.

## 5. How the LGIC Moves: The Results of Normal Mode Analyses

### 5.1. In Vacuo Simulations

With our receptor constructs now relatively whole and putative anesthetic binding sites identified, it has been our eventual goal to seek how the binding of anesthetics and other ligands may alter overall ion channel function. Despite, the vagaries of the exact location of the an anesthetic binding site, overall protein motions should be quite similar in either model due to their dependence upon the backbone structure common to both. Current computational capabilities could allow us to perform formal molecular dynamics simulations on a relatively small protein for the further examination of larger scale motions in exacting detail. However, performing large scale molecular dynamics calculations on the millisecond timescale required for actual ion channel gating on LGIC models that contain approximately 26,000 atoms each, is intractable even with the most modern computational hardware.

We have therefore resorted to the more approximate techniques of normal mode analysis to study large scale protein motion [63]. Normal mode analysis is a computational means of decomposing the whole motion of a protein into individual orthogonal vibrations. We had hypothesized that the highest amplitude, lowest frequency normal mode of these proteins should characterize the natural resonance of channel gating within a LGIC. That is, the natural opening and closing motion associated with ion passage should be represented in the highest amplitude harmonic vibration of the protein. We performed these calculations using three different methods of normal mode analysis *in vacuo* and our hypothesis proved to be correct.

### 5.2. Simulations of an LGIC in the Fully Hydrated Lipid Bilayer

**Figure 7:**
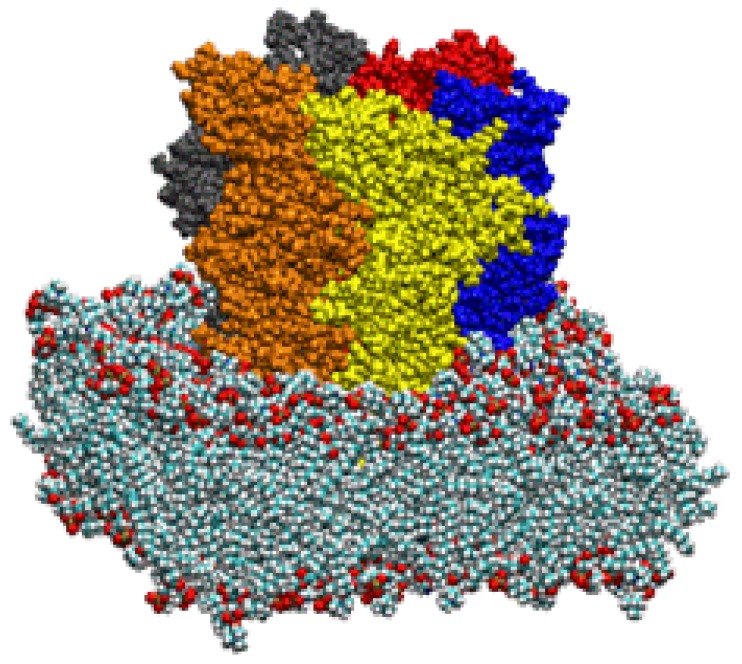
Side view of a ligand gated ion channel model in CPK atomic format suspended in a lipid bilayer. Each protein subunit is individually colored.

With the advent of greater computing horsepower, we have gone on to show the successful application of an elastic network calculation on our previously published model of a glycine alpha one receptor (GlyRa1), further suspended in a fully hydrated lipid bilayer. Despite the presence of over 100,000 atoms, these calculations continue to demonstrate a symmetric motion of the ion channel protein that is consistent with the gating motion demonstrated for previous *in vacuo* work by our group and others (Figure 7) [64]. We started by using the coordinates of the GlyRa1 model from our previous work. A 100 x 100 Å lipid bilayer matrix was constructed from POPC and then hydrated on both surfaces with water molecules using the VMD 1.86 software package (NCSA, Urbana, IL, USA). Discovery Studio 1.7 (Accelrys) molecular modeling software was used to insert our GlyRa1 model into the lipid bilayer such that the known interfacial residue GLY 221 was at the POPC-water interface. All waters within 3.8 Å of the protein were removed as were all lipid molecules within 2 Å of the protein. Hydrogens were added followed by energy minimization of the entire system to remove energetically unfavorable contacts. The system was subsequently further hydrated within the GROMACS software suite and subjected to further energy equilibration via molecular dynamics simulation with periodic boundary conditions. Normal mode analysis was performed using an all atom elastic network model developed by Lindahl which takes advantage of a sparse matrix implementation for computational efficiency. Despite the large size of the system, the introduction of water and lipid did not grossly distort the overall gating motion of the GlyRa1 noted in previous works. Normal mode analysis revealed that the GlyRa1 in a fully hydrated bilayer environment continues to demonstrate an iris-like gating motion as a low frequency, high amplitude natural harmonic vibration. Furthermore, the introduction of periodic boundary conditions allowed simultaneous harmonic vibrations of lipid in sync with the protein gating motion that are compatible with reasonable lipid bilayer perturbations. This was among the first description of a normal mode calculation describing large-scale protein dynamics and ion channel gating in the presence of a fully hydrated lipid bilayer complex. This analysis was only possible on such a large system due to the computational efficiencies of the elastic network approximation.

### 5.3. Implications

**Figure 8:**
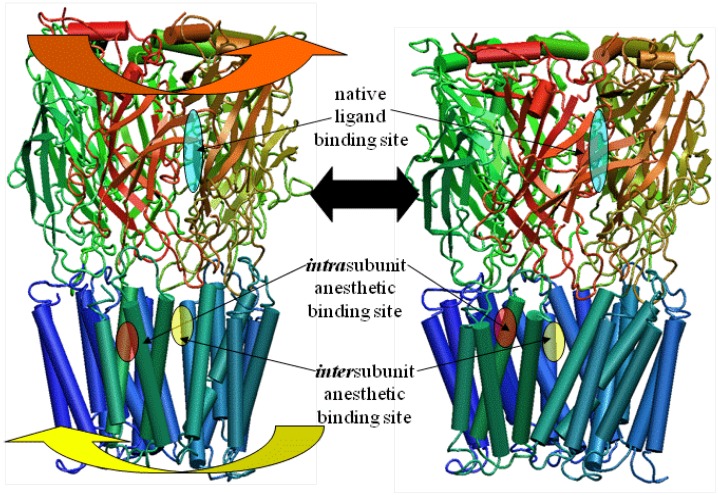
Side view of a LGIC with gating motion illustrated, as one might wring water from a washcloth in opposite directions when gripping from opposite ends.

These calculations demonstrate a great many characteristics of LGIC motion and have many implications for future study. It can be shown that one of the most fundamental motions of our LGIC models is characterized by an “iris-like” wringing motion (Figure 8).

This motion produces an alteration in pore diameter in the general direction of ion channel opening. The frequency of such motion appears to occur on the subnanosecond time scale. While this seems a bit faster than the experimentally derived time constant for ion channel gating, this time scale implies that the ion channel is vibrating in the general direction of channel gating on a virtually continuous basis. However, it is probably not open long enough, on the average, for actual ion transduction. It would not be until a large enough random thermal event occurred that an increase in amplitude or time spent in the open state would cause spontaneous channel opening to occur. This likelihood of opening is apparently increased by the presence of native ligand. It is further modulated by the presence of general anesthetics. In the case of the cation conducting nAChR, general anesthetics inhibit the excitatory currents due to acetylcholine binding. On the other hand, general anesthetic binding in GlyRa1 and GABAR potentiates the anionic inhibitory currents induced by glycine or GABA binding respectively.

A plot of the backbone deviations during motion of the residues known to modulate anesthetic effects when mutated demonstrates that putative anesthetic binding pockets from any of the aforementioned models seem to be consistently located in a region of intermediate motion. This may prove to be some critically important “hinge point” within the complex. These cavities may also have a transmembrane/boundary mode of access. Anesthetic binding may actually alter the overall motion of a ligand-gated ion channel by a “foot-in-door” motif, resulting in the altered likelihood of and/or time spent in the open channel state. 

Finally, it appears that the transduction of native ligand binding from the extracellular LBD to the TMD occurs via two methods of coupling: The first method is by way of the protein backbone, as the amino acid sequence transitions from the LBD to the TMD. The second is through the intercalation of the TMD2-3 loop with loops 2 and 7 from the LBD. 

## 6. Conclusion and Future Directions

For the last one and a half decades, anesthetics have been given both safely and effectively for the relief of patient suffering. While a great many theories have been put forth, it has become increasingly apparent that a significant focus of anesthetic action lies within the family of ligand-gated ion channels (LGICs). These channels have a transmembrane region that is composed of a pentamer of four-helix bundles along with their putative amphiphilic anesthetic binding sites. This amphiphilic nature may help to explain the need for hydrophobicity expressed in the Meyer-Overton correlation, as well as the need for hydrophilicity required by the exceptions to the Meyer-Overton rule. The overall motion of these channels is consistent with the natural harmonic vibrations demonstrated by normal mode analyses. While still the goal of future pursuits, anesthetic binding may alter the overall motion of a ligand-gated ion channel by a “foot-in-door” motif, resulting in the altered likelihood of ion channel conductance when in the presence of the native ligand. Despite the great distance from the native ligand binding site to the gating elements of the pore, such analysis also demonstrates a ready means of information transduction over a large span. In the future, such calculations may allow for the analysis of subtle effects from mutations and small ligand binding. In addition, such a model may also be used to illustrate actual protein dynamics and more complete gating cycles elucidated via large-scale molecular dynamics and normal mode calculations. These latter calculations will become ever more possible as the huge computing horsepower requirements become more readily attainable. Especially in the case of molecular dynamics simulations, more realistic analyses will likely include the direct effects of explicit lipid moieties, water, and the binding of ligands in multiple portions of the ion channels. Furthermore, dynamic analyses will also allow the potential simulation of ion channel flux parameters. Finally, the ion channel motion that is associated with gating may actually one day serve as additional endpoints for future drug design.
